# When Usability Isn’t Enough: Understanding Rural Challenges in Respiratory Virus Test Interpretation

**DOI:** 10.3390/diagnostics16142275

**Published:** 2026-07-21

**Authors:** Jennifer K. Frediani, Cheryl L. Stone, Sarah Farmer, Amanda Peagler, Richard Parsons, Adrianna L. Westbrook, Laura M. Johnson, Mark A. Griffiths, Wilbur A. Lam, Greg S. Martin

**Affiliations:** 1 Nell Hodgson Woodruff School of Nursing, Emory University, Atlanta, GA 30322, USA; richard.parsons@emory.edu; 2 Children’s Healthcare of Atlanta, Atlanta, GA 30329, USA; cheryl.stone@choa.org (C.L.S.); mark.anthony.griffiths@emory.edu (M.A.G.); 3 Center for Advanced Communications Policy, Georgia Institute of Technology, Atlanta, GA 30308, USA; sarah.farmer@gtri.gatech.edu (S.F.); amanda.peagler@gtri.gatech.edu (A.P.); 4 Atlanta Center for Microsystems-Engineered Point-of-Care Technologies, Emory University, Atlanta, GA 30322, USA; 5 Department of Pediatrics, Emory University School of Medicine, Atlanta, GA 30322, USA; adrianna.lynn.westbrook@emory.edu (A.L.W.);; 6 Wallace H. Coulter Department of Biomedical Engineering, Georgia Institute of Technology, Emory University, Atlanta, GA 30332, USA; 7 Aflac Cancer and Blood Disorders Center of Children’s Healthcare of Atlanta, Atlanta, GA 30322, USA; 8 Department of Pulmonary, Emory University School of Medicine, Atlanta, GA 30322, USA

**Keywords:** Rural Health, at-home diagnostics, respiratory virus, usability

## Abstract

**Background/Objectives**: This study aimed to evaluate the usability and comprehension of at-home respiratory diagnostic tests for COVID-19 and influenza among rural and urban populations in Georgia. It sought to identify disparities in test interpretation and outcomes that could inform public health interventions tailored to underserved communities. **Methods**: Participants with respiratory symptoms (*N* = 592) were enrolled through community events in three rural Georgia counties (*N* = 188) and urban clinics in Atlanta (*N* = 404). Structured usability assessments and RT-PCR-confirmed diagnostic testing were conducted. Demographic, clinical, and survey data were analyzed using *t*-tests, z-tests, and Fisher’s exact tests to compare rural and urban participants. **Results**: Rural participants were older and more likely to be White. Urban participants had significantly higher infection positivity rates for COVID-19 (18.5% vs. 10.2%) and Flu A (29.2% vs. 1.8%). While overall usability ratings were high across both groups (>94%), rural participants had significantly lower odds of identifying invalid test results and were more likely to continue using tests after procedural errors. **Conclusions**: Despite strong usability ratings, diagnostic test comprehension differed by geography. Rural participants demonstrated notable gaps in recognizing invalid results, underscoring the need for targeted educational strategies and simplified instructions to ensure equitable test interpretation and public health response.

## 1. Introduction

Rural communities in Georgia have faced disproportionately high burdens from respiratory illnesses such as COVID-19 and influenza, exacerbated by socioeconomic challenges and healthcare disparities. Notably, rural Georgians have historically experienced influenza and pneumonia-related death rates over 2.5 times higher than those in metropolitan counties. For instance, Miller County, with only 5700 residents, reported a flu-related mortality rate four times greater than Georgia’s urban counties over the past decade [1,2]. During the COVID-19 pandemic, these disparities further intensified: as of March 2025, rural communities reported 767 COVID-19 infections and 39 COVID-19-related deaths per 100,000 residents, significantly higher than urban counterparts (612 infections and 23 deaths per 100,000) [3]. Importantly, rural counties with significant Black populations, such as Hancock, Randolph, and Terrell, ranked among the highest nationally in COVID-19 death rates [4,5].

Health disparities in rural Georgia reflect national trends but occur at higher rates, particularly in preventable conditions like heart disease and chronic lower respiratory diseases. In 2022, preventable heart disease deaths were more prevalent in rural areas (56%) than in urban regions (41%), exacerbated by limited access to emergency care and the lingering effects of COVID-19 [6,7]. Additionally, Georgia’s rural regions, situated within the “Stroke Belt,” experience disproportionately high stroke rates due to risk factors such as hypertension, smoking, and limited healthcare access. Despite the widespread availability of home diagnostic tests during the COVID-19 pandemic, limited research has explored how rural populations interact with these tools under real-world conditions [8]. Understanding these interactions is critical for developing more intuitive, error-tolerant diagnostic technologies that accommodate the unique constraints of rural settings, such as limited broadband access, lower health literacy, and reduced access to follow-up care. Addressing these disparities requires targeted public health interventions that prioritize rural voices in diagnostic research, as their perspectives are crucial for identifying usability challenges in testing procedures. Rural populations may encounter unique barriers in recognizing invalid test results and appropriately responding to testing errors, emphasizing the need for community-driven educational initiatives and tailored communication strategies to ensure interventions are culturally relevant and effective.

At-home medical tests have increased over the last decade in both options available and utilization by lay people. Early on during the pandemic, some scientists were cautious of widespread at-home COVID-19 test usage. The ethical concerns included accuracy of the test results, misinterpretation of test results, privacy, and fair allocation of scarce resources, but little was known about how these tests would be used throughout the pandemic and how they were evaluated [9]. Many older adults (48%) have purchased a home test of some kind, and 32% of those were a COVID-19 test and they planned to use them [10].

The pandemic further accelerated the decentralization of diagnostics, with nearly one billion at-home, over-the-counter COVID-19 tests produced monthly by February 2022 [11]. While the increased capacity to test undoubtedly saved lives, with one model suggesting up to 1.4 million lives were saved [11], this expansion of diagnostics into the home also comes with unique challenges. Home tests present new difficulties surrounding clinical, performance, regulatory, and usability barriers [12]. Human factors considerations are progressively pertinent in this use case of novice home users in environments that often contain distractions and suboptimal conditions [13]. Ensuring the design of tests and instructional materials are optimized and accessible to home user populations is key.

Home-based, over-the-counter lateral flow assays (LFAs) represent crucial solutions for bridging persistent testing gaps, enabling prompt identification of infections, and reducing disease transmission in isolated communities. Given rural residents’ unique challenges, such as lengthy distances to healthcare facilities, limited local availability of home tests, delayed shipping, financial barriers, and lower health literacy, the usability of these at-home tests remains paramount. Clear and accessible instructions minimize user error opportunities, and easily interpretable results are critical to ensuring the diagnostic effectiveness and subsequent healthcare decisions made by rural populations.

In response to these critical gaps, the Rapid Acceleration of Diagnostics (RADx) initiative [14] was established during the pandemic to rapidly develop and scale diagnostic testing technologies, initially focusing on SARS-CoV-2 and subsequently expanding to other respiratory pathogens. Currently, five years after the peak of the pandemic, numerous diagnostic options are widely available. However, questions remain regarding whether these diagnostics are effectively utilized and interpreted correctly by populations facing significant health barriers. The RADx Tech program at Emory University continues to evaluate and improve point-of-care testing strategies, focusing particularly on underserved rural communities.

This study aimed to evaluate the usability and results interpretation of widely available home respiratory diagnostic tests for COVID-19 and multiplex tests that include influenza A within underserved rural communities, using urban communities in the Atlanta metropolitan area for comparison. Partnering with local health authorities and community leaders, we established testing sites in three rural Georgia counties—Blairsville (Union County), Dublin (Laurens County), and Sandersville (Washington County) alongside comparative testing conducted in Atlanta metro areas.

Ultimately, addressing these unique barriers through targeted improvements in diagnostic test usability represents a critical ongoing strategy to reduce health disparities and enhance public health outcomes in Georgia’s underserved rural regions.

## 2. Materials and Methods

### 2.1. Clinical Protocol for RADx

The Atlanta Center for Microsystems Engineered Point-of-Care Technologies (ACME-POCT) network utilized hospital and community-based clinics in urban areas and large parking lots in rural areas to enroll participants. There were three rural events: the first in August–September 2021 and the second and third in February 2025. Eligible participants were identified consecutively at each study site. Inclusion criteria for the parent study included any person with upper respiratory infection symptoms seeking testing. Exclusion criteria for the parent study included patients who were unable to tolerate a nasal swab or were unable to provide informed consent. Following identification, eligible participants for the parent study were approached by study personnel, who obtained informed consent. Clinical electronic case report forms were used to collect demographic and clinical variables, including age, sex, race, ongoing medical conditions, upper respiratory infection-related symptoms and date of symptom onset, SARS-CoV-2 exposure, prior infection, and vaccine status. Vaccine status for influenza was added to the database after the first rural event. Clinical and demographic variables were collected in a centralized, web-based database (REDCap, Nashville, TN, USA). The study protocol was approved by the Emory Institutional Review Board, Children’s Healthcare of Atlanta, and the Grady Research Oversight Committee (IRB#00001082). For all participants, an FDA-approved (through Emergency Use Authorization) reverse transcription polymerase chain reaction (RT-PCR) SARS-CoV-2 test was administered within 24 h of study enrollment (anterior nasal swab sample collected with a flocked swab [PurFlock Ultra^®^ Dry Transport System 25-3606-U BT] and diluted in 3.0 mL saline). Participant samples from the time period of the first rural event were analyzed by reverse transcription polymerase chain reaction on one of the following: Abbott Alinity M, Abbott m2000 (Abbott, Des Plaines, IL, USA) Panther Fusion (Hologic, Marlborough, MA, USA), Roche Cobas 6800 (Roche, Basel, Switzerland), or Cepheid GeneXpert Xpress (Cepheid, Sunnyvale, CA, USA). Further details are given in Supplemental Table S1.

### 2.2. Rural Cohort: Community Engagement and Site Logistics

To evaluate usability of at-home respiratory diagnostic tests in underserved rural populations, we partnered with local leaders and health agencies in three Georgia communities: Blairsville (Union County), Dublin (Laurens County), and Sandersville (Washington County). These counties were selected for their limited healthcare infrastructure, elevated poverty rates, and disproportionately high respiratory illness burdens.

In Blairsville, a mountain community of approximately 650 residents (24,000 county-wide) [15,16], access to care is limited to a single 42-bed hospital. During a COVID-19 surge in August 2021, the community experienced a 30% positivity rate with no available in-clinic or over-the-counter testing options, and up to 10-day result delays for samples shipped to Atlanta. Following a request from a local school leader, our team mobilized a rapid response testing initiative in coordination with county and city officials, healthcare leadership, and public safety. A temporary drive-through testing site was established at the local farmers’ market, chosen for its accessibility, shelter, electricity, internet, and refrigeration capacity. Research and clinical samples were collected onsite, with clinical testing routed to partner laboratories in Atlanta to mitigate local lab capacity limitations.

In Dublin, a community of 16,000 with a 19-bed hospital, we collaborated with the Georgia Rural Health Innovation Center and the South-Central Health District (District 5-1). Dublin’s poverty rate (22.6%) and limited high school attainment (16.1% without diploma) reflect systemic healthcare access challenges [17]. The Georgia Department of Public Health’s Emergency Preparedness Office assisted with site setup and operations, securing a local church with a covered drive-through structure and essential utilities for a two-day testing event, enrolling 54 participants with respiratory symptoms.

In Sandersville, with a population under 6000 and a local hospital of 56 beds, testing access was primarily limited to a free rapid kiosk. With 26.7% of residents living in poverty and over 21% without a high school diploma, the community also experienced a high respiratory illness mortality rate (682 per 100,000 between 2020–2025) [18]. Through coordination with the local County Health Department, our team conducted a two-day drive-through testing event behind the health department building, enrolling 59 participants with respiratory symptoms.

Across all sites, partnerships with local institutions were essential to navigating logistical challenges, identifying secure and accessible testing locations, and building trust in communities historically underserved by public health infrastructure.

### 2.3. Urban Cohort

The continuous RADx study effort was conducted in the Atlanta metropolitan area starting in June 2020 through March 2027. Testing sites ranged over the years from outdoor drive through testing sites to hospitals and urgent care clinics. Atlanta’s population as of the last census in July 2024 was 520,070 within the city limits [19] and the 6th largest metropolitan area in the U.S. at 6,482,182 residents. As of 24 March 2024 there were a total of 9412 deaths from COVID-19 in the core counties of metro Atlanta (total population 3,958,906) [20]. The urban cohort includes individuals who were tested during the same windows of the rural recruitment events (1 August 2021–30 September 2021; 27 January 2025–28 March 2025), resulting in enrolling 404 participants with respiratory symptoms.

### 2.4. Statistics

Inclusion criteria for this analysis includes all participants who were enrolled within the time frame of 4 weeks prior to and 4 weeks after each of the following three rural events: Blairsville, Dublin Outreach, and Sandersville Outreach. Exclusion criteria for this analysis include all participants who did not complete enrollment in the study.

The data was split into two cohorts based on enrollment location: rural (defined as all participants enrolled in one of the Blairsville, Dublin Outreach, or Sandersville Outreach locations) and urban (defined as all other participants enrolled in the study at large). Summary tables were constructed based on demographic (age, race, education level, etc.) and clinical (comparator results and vaccine status for COVID-19 and Influenza A) variables as well as for each response to the Independent Test Assessment Program (ITAP) [21] questionnaires provided to participants after enrolling with an ITAP device. The ITAP was within the RADx infrastructure and aimed to accelerate review and evaluation of diagnostic tests for various infectious diseases. The usability questionnaire included four Likert-type scale questions designed to assess subjective ease of use, as well as a series of questions to evaluate comprehension of the test protocol and instructions. Due to seasonal variability in respiratory viruses, we conducted a sensitivity analysis of positivity/CT by cohort, stratified by recruitment window (#1 August–September 2021; #2 January–March 2025).

For all variables with quantitative measures (age, number of previous home tests, etc.), bivariate logistic regressions were performed. For all categorical variables, Fisher’s exact tests were performed. *p*-values from all tests are provided in the summary tables, as well as odds ratios and corresponding 95% confidence intervals (CIs).

## 3. Results

Participants differed between rural and urban areas in several key characteristics. Rural participants tended to be older and more likely to identify as White. Educational attainment did not significantly differ between the two groups when comparing each category to the reference group of those without a high school diploma (Table 1).

Infection positivity rates varied significantly between rural and urban participants. Urban participants were more likely to test positive for COVID-19 compared to those in rural areas (18.5% vs. 10.2%), and Flu A positivity was notably higher among urban participants (29.1% vs. 1.8%) (Table 2). Rural participants were more likely than urban participants to report previous home test use (78.8% vs. 66.0%). Regarding vaccination, rural participants reported lower rates of COVID-19 vaccination compared to urban participants (9.3% vs. 29.8%). No statistically significant differences were observed between groups for influenza A vaccination (Table 2). Stratified comparisons by recruitment cohort can be found in Supplemental Table S2.

Participants from both rural and urban settings reported high levels of usability for at-home diagnostic tests, with over 94% in each group agreeing that instructions were easy to follow, sample collection was easy to perform, and results were easy to interpret. There were no significant differences between groups in the average number of times participants had previously used a home test. Rural participants had 3.06 times the odds (95% CI: [1.32, 7.32]; *p* = 0.005) of reporting that they would continue with the current test and accept current results after skipping a step or performing a test incorrectly compared to urban participants. Differences also emerged in test comprehension: the odds of indicating that a test lacked control or test lines for rural participants was lower than the odds of indicating that a test lacked control or test lines for urban participants (OR [95% CI] 0.32 [0.14, 0.68]; *p* = 0.001), while the odds of identifying the absence of both a control line and a test line as a marker of invalid results for rural participants was higher than the odds for urban participants (2.26 [1.11, 4.64]; *p* = 0.018). Both groups had over 95% of participants correctly associate appropriate symptoms, such as fever and fatigue, with test use. While most participants recognized that a positive result should prompt self-isolation, a small number in each group selected less-than-optimal actions following a negative result, such as not isolating or retesting. Research staff confirmed valid test results for >96% participants in each cohort, with no discrepancies observed between participant and staff interpretations (Supplemental Table S3).

## 4. Discussion

This study identified significant differences between rural and urban participants regarding infection positivity rates, vaccination status, and test usability. Urban participants exhibited notably higher infection positivity rates for both COVID-19 and Flu A, with nearly twice the COVID-19 positivity rate of rural participants and a significantly higher Flu A positivity rate. Additionally, rural participants reported lower rates of COVID-19 vaccination more than one year prior to study visit compared to urban participants. Despite these disparities, over 94% of participants in both settings reported subjective ease of use for at-home diagnostic tests, indicating that following test instructions, collecting and applying sample, and interpreting results were easy to do. However, differences in test comprehension emerged, with rural participants less likely to recognize when a test was invalid due to missing control lines and more likely to continue using a test after skipping a step.

The differences in infection positivity rates between rural and urban participants may be attributed to varying levels of population density, healthcare access, and adherence to preventive measures. The lower vaccination rates among rural participants could reflect misinformation or logistical barriers to vaccine access. The discrepancies in test comprehension may stem from differences in health literacy, prior diagnostic test experience, and local public health messaging, with rural participants potentially having less exposure to testing protocols.

Diagnostic testing usability in rural versus urban populations has not been discussed in the literature to date. The observed disparities align with previous studies demonstrating that rural populations often report lower infection rates but face challenges in health literacy and diagnostic test comprehension. The higher COVID-19 positivity rate in urban settings may reflect greater population density, increased exposure risk, or more robust testing infrastructure. A study conducted in a neighboring Southern state found that the distribution of healthcare facilities between urban and rural spaces are unequal and that almost 2000 health centers, mostly serving low-income and racial/ethnic minority populations, temporarily closed in early 2020, which limited access to primary care services, and thus disproportionately affected access to care for marginalized and disadvantaged populations [22]. This is despite the increased odds of mortality from COVID-19 in rural communities [23].

In contrast, the lower vaccination rates in rural areas may suggest that recent public health campaigns did not effectively reach these populations, and the historical vaccine hesitancy in rural settings continue, especially in the rural South [24]. This corroborates a previous study conducted from data from the National Health and Aging Trends Study, that found rural residents are less likely to perceive COVID-19 as a severe threat [25]. Callaghan and colleagues found that rural residents were also less likely to have worn masks, sanitized their home or workplace, avoided dining in restaurants or worked from home [26].

The findings highlight the importance of continued efforts to include rural populations in diagnostic testing usability evaluations, particularly given the observed differences in determining test validity. While the pandemic era has subsided, understanding how rural participants use and interpret at-home diagnostic test instructions remains critical for future public health preparedness. Educational campaigns that emphasize the recognition of invalid test results and the steps to take following a testing error could mitigate potential misinterpretations [27].

Test developers should use strategies to increase the usability for results interpretation, particularly create instructions for use that are accessible to populations with varying levels of health literacy, with a particular focus on interpreting test results [28]. Results interpretation should guide users through the process by first presenting invalid results, followed by negative results, and lastly, positive results. These sections should include images of all possible results, including various combinations for multiplex tests. Additionally, providing instructions in alternate languages and in multiple modalities will increase the accessibility. Ensuring instructions for use are accessible for various populations may also improve diagnostic accuracy and overall health outcomes.

Other strategies to improve results interpretation would be to change the results presentation on the test device. Ideally, tests would present users with full words, such as “invalid,” “positive,” or “negative,” as well as using full virus names. For non-digital tests, full-word labeling should be used on the device itself to prevent confusion. For example, the word “control” would prevent users from mistaking a “c” label for “COVID”. Full-word labeling for virus names is additionally helpful for multiplex result interpretation.

The study’s strengths include its comparative design, inclusion of multiple infection types, and comprehensive assessment of both diagnostic test use and comprehension in both rural and urban settings. However, the study has several limitations, including the reliance on self-reported data, which may introduce recall or response biases. The sample size, though substantial, may not fully capture the diversity of rural and urban populations, particularly regarding socioeconomic status and access to healthcare. We did not collect information on insurance status in this study and therefore cannot make any conclusions on healthcare access. The rural settings were chosen because of contacts made through the RADx program and did not include a random sampling of rural settings in the state of Georgia.

Future research should prioritize the inclusion of rural populations in diagnostic usability studies, recognizing their critical role in identifying potential gaps in test comprehension and validity interpretation. Future usability studies should be more comprehensive in scope and include error investigation in order to pinpoint the causes of use errors. Given the observed differences in recognizing invalid test results, rural participants provide valuable insights that can inform the development of more effective test instructions and educational interventions. Additionally, assessing how tailored health communication strategies resonate with rural populations may further illuminate ways to optimize diagnostic accuracy and mitigate health disparities. Longitudinal studies focusing on the sustained impact of educational interventions in rural settings could also provide important evidence for refining public health messaging and testing protocols [29].

## 5. Conclusions

In conclusion, this study highlights important disparities in infection positivity rates, potential health care access, vaccine uptake, and diagnostic test comprehension between rural and urban populations. While overall, participants reported that tests were easy to use, specific areas of misunderstanding were identified, particularly regarding test validity and appropriate follow-up actions. Public health efforts to enhance diagnostic test literacy, particularly in rural settings, and to address gaps in vaccination coverage in urban areas may reduce infection rates and improve health outcomes across both populations.

## Figures and Tables

**Table 1:** Demographic differences between rural and urban populations.

Variable	Rural (*N* = 188)	Urban (*N* = 404)	Odds Ratio (95% CI)	*p*-Value
Age (mean [SD]) (years)	50.8 (16.7)	30.3 (20.6)	1.05 (1.04, 1.06)	**<0.001**
Missing	*0*	*0*		
Race (n [%])				
White	1134 (60.1%)	165 (40.8%)	Ref	
Black/African American	69 (36.7%)	197 (48.8%)	0.51 (0.35, 0.75)	**<0.001**
Asian	1 (0.5%)	19 (4.7%)	00.8 (0.00, 0.50)	**0.001**
Other	1 (0.5%)	10 (2.5%)	0.15 (0.00, 1.06)	0.05
Missing	0	*0*		
Ethnicity (Hispanic/Latino)	6 (3.2%)	55 (13.8%)	0.2 (0.07, 0.49)	**<0.001**
	*1*	*4*		
Education Level				
No high school diploma	6 (6.5%)	13 (10.0%)	Ref	
High school diploma	37 (39.8%)	48 (36.9%)	1.66 (0.53, 5.86)	0.442
Some college, no degree	24 (25.8%)	36 (27.7%)	1.44 (0.43, 5.28)	0.596
Bachelor’s or Associate’s degree	22 (23.7%)	20 (15.4%)	2.35 (0.67, 9.07)	0.170
Graduate degree	2 (2.2%)	7 (5.4%)	0.63 (0.05, 4.93)	>0.999
Other	2 (2.2%)	6 (4.6%)	0.73 (0.06, 5.97)	>0.999
Missing	*95*	*274*		
Income Level				
Less than $15,000	19 (16.8%)	26 (12.4%)	Ref	
$15,001–$45,000	37 (32.7%)	41 (19.6%)	1.23 (0.55, 2.78)	0.707
$45,001–$90,000	19 (16.8%)	35 (16.8%)	0.75 (0.3, 1.81)	0.536
>$90,000	19 (16.8%)	32 (15.3%)	0.81 (0.33, 2.00)	0.679
Deferred	19 (16.8%)	75 (35.9%)	0.35 (0.15, 0.81)	**0.008**
Missing	*75*	*711*		
Medical Conditions				
Hypertension	75 (39.9%)	63 (15.6%)	Ref	
Diabetes	41 (21.8%)	27 (6.7%)	1.27 (0.68, 2.41)	0.457
Obesity	26 (13.8%)	19 (4.7%)	1.15 (0.55, 2.42)	0.732
Heart Disease	18 (9.6%)	13 (3.2%)	1.16 (0.49, 2.8)	0.842
Chronic Lung Disease	9 (4.7%)	10 (2.5%)	0.76 (0.25, 2.22)	0.629
Chronic Kidney Disease	4 (2.1%)	7 (1.7%)	0.48 (0.1, 2.00)	0.349
Chronic Liver Disease	4 (2.1%)	2 (0.5%)	1.67 (0.23, 19.08)	0.69
Missing	*0*	*0*		

*p*-values indicate significance.

**Table 2:** Infection status differences between rural and urban populations.

Variable	Rural (*N* = 188)	Urban (*N* = 404)	OR (95%) CI)	*p*-Value
COVID-19 positivity	19 (10.2%)	73 (18.5%)	0.49 (0.27, 0.86)	**0.010**
COVID-19 Ct value	10.0 (13.8)	18.0 (12.5)	0.95 (0.93, 0.98)	**<0.001**
Missing	*2*	*9*		
Flu A positivity	2 (1.8%)	61 (29.2%)	0.04 (0.01, 0.18)	**<0.001**
Flu A1 Ct value *	25.2 (0.7)	26.2 (5.7)	0.97 (0.71, 1.24)	0.796
Flu A2 Ct value *	26.1 (0.7)	27.5 (5.7)	0.98 (0.85, 1.22)	0.780
Missing	77	195		
Taken a home test previously	89 (78.7%)	138 (66.0%)	1.9 (1.09, 3.41)	**0.021**
Missing	*75*	*195*		
COVID-19 Vaccine Status				
None	60 (40%)	188 (60.3%)	Ref	
Vaccinated > 1 year prior to study visit	14 (9.3%)	93 (29.8%)	0.47 (0.23, 0.91)	**0.022**
Vaccinated ≤ 1 year of study visit	12 (8.0%)	18 (5.7%)	2.08 (0.86, 4.88)	0.077
Vaccinated but dose date uncertain	64 (42.7%)	13 (4.2%)	15.27 (7.68, 32.38)	**<0.001**
Missing	*38*	*92*		
Influenza Vaccine Status				
None	60 (53.1%)	133 (63.6%)	Ref	
Vaccinated >1 year prior to study visit	0 (0%)	4 (1.9%)	0 (0, 3.46)	0.316
Vaccinated ≤ 1 year of study visit	28 (24.8%)	65 (31.1%)	0.96 (0.53, 1.68)	0.892
Vaccinated but dose date uncertain	25 (22.1%)	7 (3.36%)	7.84 (3.08, 22.68)	**<0.001**
Missing	*75*	*195*		

Values reported are n (%) and Mean (SD). * The FluA assay provides two Ct values; they both correspond to PA and PB2 targets and are thus labeled FluA1 and FluA2. *p*-values indicate significance.

## Data Availability

The data presented in this study are available on request from the corresponding author due toreasons of sensitivity. Data are located in controlled access data storage at Emory University.

## Supplementary Materials

The following supporting information can be downloaded at: https://www.mdpi.com/article/10.3390/diagnostics16142275/s1, Table S1. Commercial Assays and Sample Types for Recruitment Windows. Table S2. COVID-19 and Flu A Positivity and Ct Values by Cohort, Stratified by Recruitment Window. Table S3. Usability question differences between rural and urban populations.

## Abbreviations

The following abbreviations are used in this manuscript:

LFA	Lateral flow assay
RADx	Rapid Acceleration of Diagnostics
SARS-CoV-2	Severe Acute Respiratory Syndrome coronavirus 2
ACME-POCT	Atlanta Center for Microsystems Engineered Point-of-Care Technologies
RT-PCR	Reverse transcription polymerase chain reaction
ITAP	Independent Test Assessment Program
CI	Confidence interval
OR	Odds ratio
