# Supplementary material for: When Usability Isn’t Enough: Understanding Rural Challenges in Respiratory Virus Test Interpretation
Source: Diagnostics (Basel). 2026 Jul 21;16(14):2275. doi: 10.3390/diagnostics16142275 (PMC13408658; doi:10.3390/diagnostics16142275)
Supplement: Supplementary file 1 [file diagnostics-16-02275-s001.zip › diagnostics-4265633-supplementary.pdf]

Supplemental Table S1. Commercial Assays and Sample Types for Recruitment Windows

Location	Dates	Sample type	Diagnostic test evaluated	Commercial Assay	# of tests conducted
Blairsville (Rural)	8/1/21-9/31/21	NP	M2Dx – 2200 (n=75); Mologic (n=1)	Abbott Alinity M (Abbott, Des Plaines, IL)	75
Atlanta Metro (Urban)	8/1/21-9/31/21	NP (n=107), AN (n=24)	Mesa-Accula (n=49), M2Dx – 2200 (n=24), Salignostics (n=29), QooLabs (n=24), Oceanit (n=5), Oasis (n=2)	Abbott Alinity M	131
		NP	Mesa-Accula	Abbott m2000	1
		NP	Salignostics (n=12)	CEPHEID (multiplex) (Cepheid, Sunnyvale, CA)	12
		NP (n=1), AN (n=4)	M2Dx – 2200 (n=5)	CEPHEID Xpert (Cepheid, Sunnyvale, CA)	5
		NP	M2Dx – 2200 (n=11), Salignostics (n=4), Qoolabs (n=7), Oceanit (n=13)	Roche Cobas 6800 (Roche, Basel, Switzerland)	32
				CEPHEID (multiplex) (Cepheid, Sunnyvale, CA)	364
Dublin and Sandersville (Rural)	1/27/25-3/28/25	AN	Genbody [COV I FLA I FLB] (n=113)	CEPHEID (multiplex) (Cepheid, Sunnyvale, CA)	113
Atlanta Metro (Urban)	1/27/25-3/28/25	AN (n=206), MT (n=3)	Watmind [COV I FLA I FLB] (n=7), Meridian [COV I FLA I FLB] (n=53), COVI-Go [COV I FLA I FLB] (n=22), Maxim [COV I FLA I FLB] (n=51), Genbody [COV I FLA I FLB] (n=16), Status [COV I FLA I FLB] (n=50), Abbott BinaxNOW [COV Only] (n=10)	CEPHEID (multiplex) (Cepheid, Sunnyvale, CA)	209

Supplemental Table S2. COVID-19 and Flu A Positivity and Ct Values by Cohort, Stratified by Recruitment Window

	Recruitment Window #1 (8/1/21-9/30-21)				Recruitment Window #2 (1/27/25-3/28/25)		
	Rural (N=75)	Urban (N=195)	p-value		Rural (N=113)	Urban (N=209)	p-value
COVID-19 positivity	13 (17.3%)	53 (28.5%)	0.059		6 (5.4%)	20 (9.6%)	0.282
COVID-19 Ct value	25.3 (8.8)	23.3 (7.2)	0.395		4.5 (10.8)	11.7 (14.4)	0.019
Missing	0	9			2	0	
Flu A positivity	0	0	--		2 (1.8%)	61 (29.2%)	<0.001
Flu A1 Ct value*	--	--	--		25.2 (0.7)	26.24 (5.7)	0.796
Flu A2 Ct value*	--	--	--		26.1 (0.7)	27.5 (5.7)	0.780
Missing	--	--	--		2	0	

**Bolded results represent significance

Supplemental Table S3. Usability question differences between rural and urban populations

Variable	Rural (N=188)	Urban (N=404)	OR (95% CI)	p-value
How many times have you conducted a home test? [Mean (SD)]	5.2 (11.3)	6.3 (11.2)	0.99 (0.95, 1.02)	0.484
The overall instructions for use were clear and easy to follow.				
Strongly disagree or Disagree	2 (1.8%)	3 (1.5%)	1.2 (0.1, 10.64)	1
Neither agree or disagree	2 (1.8%)	7 (3.5%)	0.52 (0.05, 2.77)	0.501
Strongly agree or Agree	105 (96.3%)	189 (95.0%)	Ref	
The sample collection for the test was easy to perform.				
Strongly disagree or Disagree	2 (1.8%)	1 (0.5%)	3.64 (0.19, 216.57)	0.290
Neither agree or disagree	2 (1.8%)	6 (3.0%)	0.61 (0.06, 3.49)	0.718
Strongly agree or Agree	105 (96.3%)	192 (96.5%)	Ref	
Applying the sample to the device was easy to perform.				
Strongly disagree or Disagree	2 (1.8%)	5 (2.5%)	0.71 (0.07, 4.45)	1
Neither agree or disagree	1 (0.9%)	5 (2.5%)	0.36 (0.01, 3.26)	0.428
Strongly agree or Agree	106 (97.3%)	189 (95.0%)	Ref	
The test result was clear and easy to see.				
Strongly disagree or Disagree**	2 (1.8%)	0 (0%)	--	--
Neither agree or disagree	2 (1.8%)	5 (2.6%)	0.72 (0.07, 4.52)	1
Strongly agree or Agree	105 (96.3%)	190 (97.4%)	Ref	
What do I do if I skip a step or perform a step incorrectly?				
Continue with the current test and accept the results.	18 (16.5%)	12 (6.0%)	3.06 (1.32, 7.32)	**0.005**
Collect a new sample and use the same test device.	9 (8.3%)	19 (9.6%)	0.97 (0.37, 2.37)	1
Start over with a new test and make sure to perform all steps correctly.	82 (75.2%)	168 (84.4%)	Ref	
How do I know if I have NOT collected the sample or run the test correctly?				
When I see both the control line and the test line.	28 (25.7%)	23 (11.6%)	2.26 (1.11, 4.64)	**0.018**
When I do not see the control line.	45 (41.3%)	84 (42.2%)	Ref	
When I do not see the test line.	24 (22.0%)	22 (11.1%)	2.03 (0.97, 4.26)	0.053
Not applicable because this device does not have control/test lines.	12 (11.0%)	70 (35.2%)	0.32 (0.14, 0.68)	**0.001**
What symptoms would I have that would let me know this test is appropriate for me to use?				
I sprained my ankle.	1 (0.9%)	0 (0%)	**	**
I have a stomachache.	3 (2.8%)	2 (1.0%)	2.8 (0.32, 34.06)	0.349
I have a fever or am fatigued.	105 (96.3%)	197 (99.0%)	Ref	
What do I do if the COVID-19 test results are positive?				
I should continue my daily routine which includes running errands and socializing with others.	4 (3.7%)	3 (1.5%)	2.5 (0.42, 17.43)	0.247
I should self-isolate and see the doctor immediately and tell them I have tested positive for COVID-19.	104 (95.4%)	196 (98.5%)	Ref	

I should do nothing.**	1 (0.9%)	0 (0%)	**	**
What do I do if the test results are negative?				
I should stay home until I feel better, and I should see the doctor if I don't get better, or if I start feeling worse.	80 (73.4%)	166 (83.4%)	Ref	
I should ignore my symptoms and go about my daily routine.	14 (12.8%)	16 (8.0%)	1.81 (0.78, 4.18)	0.153
I should go to the hospital immediately, even when my symptoms are mild.	15 (13.8%)	17 (8.5%)	1.83 (0.8, 4.11)	0.116
By your (staff) assessment, did the individual get a valid test result? (% Yes)	112 (99.1%)	192 (96.5%)	4.07 (0.51, 185.5)	0.266
Did the subject need more than one test due to issues performing the test? (% Yes)	2 (1.8%)	7 (3.5%)	0.5 (0.05, 2.66)	0.496
Did the research staff interpretation of any result differ from that of the participant for any test? (% Yes)	0 (0%)	0 (0%)	-	-
Research Staff interpretation of COVID-19 test result (% Positive)	5 (4.4%)	20 (10.1%)	0.41 (0.12, 1.17)	0.085
Research Staff interpretation of Flu A test result. (% Positive)	4 (3.5%)	40 (20.1%)	0.14 (0.04, 0.41)	**<0.001**

**The calculated odds ratio for these categories was infinity, and the upper limit of the 95% CIs for these categories were infinity, so these were not reported. Bolded results represent significance.
